# *In vivo* maternal haploid induction in *Brassica juncea*

**DOI:** 10.1093/hr/uhaf094

**Published:** 2025-05-23

**Authors:** Yufan Chu, Shuang Jin, Ye Chen, Peijie Yao, Changchun Yu, Zhengjie Wan

**Affiliations:** National Key Laboratory for Germplasm Innovation & Utilization of Horticultural Crops, College of Horticulture and Forestry Sciences, Huazhong Agricultural University, 430070 Wuhan, China; National Key Laboratory for Germplasm Innovation & Utilization of Horticultural Crops, College of Horticulture and Forestry Sciences, Huazhong Agricultural University, 430070 Wuhan, China; National Key Laboratory for Germplasm Innovation & Utilization of Horticultural Crops, College of Horticulture and Forestry Sciences, Huazhong Agricultural University, 430070 Wuhan, China; Shenzhen Branch, Guangdong Laboratory of Lingnan Modern Agriculture, Key Laboratory of Synthetic Biology, Ministry of Agriculture and Rural Affairs, Agricultural Genomics Institute at Shenzhen, Chinese Academy of Agricultural Sciences, 518120 Shenzhen, China; National Key Laboratory for Germplasm Innovation & Utilization of Horticultural Crops, College of Horticulture and Forestry Sciences, Huazhong Agricultural University, 430070 Wuhan, China; National Key Laboratory for Germplasm Innovation & Utilization of Horticultural Crops, College of Horticulture and Forestry Sciences, Huazhong Agricultural University, 430070 Wuhan, China; National Key Laboratory for Germplasm Innovation & Utilization of Horticultural Crops, College of Horticulture and Forestry Sciences, Huazhong Agricultural University, 430070 Wuhan, China

Dear Editor,

*Brassica juncea*, an allotetraploid crop belonging to the *Brassicaceae* family, is a globally cultivated horticultural crop known for its nutritional value and multifaceted applications. It serves not only as a vegetable but also as a source of oil extraction and as a condiment. A significant challenge in breeding *B. juncea* is its tendency towards cross-pollination, which results in a complex genetic background [1]. Traditional methods for producing relatively homozygous inbred lines require over six generations of self-pollination through manual bagging, a process that is both time-consuming and inefficient. Given the importance of inbred lines in genetic research and crop improvement, traditional methods such as self-pollination through manual bagging are time-consuming and inefficient. These limitations have urgently prompted the search for more efficient breeding techniques.

The advent of doubled haploid (DH) technology has revolutionized plant breeding by enabling the rapid production of homozygous lines. Historically, the anther/pollen *in vitro* culture technique has been the main method for generating haploid plants. However, this approach is characterized by complex procedural steps and stringent culture conditions, which can extend the tissue culture period, often requiring a significant amount of time to develop into complete plants. In contrast, the maternal haploid induction system has been successfully applied in maize, offering a more convenient and simpler alternative. This method facilitates the generation of haploid plants by inducing the retention of maternal genetic material alone in the fertilized egg [2]. The homozygous characteristic of DH lines makes them extremely valuable for genetic research and efforts to enhance crop quality. The cloning of haploid induction genes, such as *MTL* and *DMP* in maize, has propelled the expansion of haploid induction systems in plant breeding [3, 4]. Notably, the *DMP* exhibits a high degree of conservation across dicotyledonous plants [5], suggesting its potential for gene-editing approaches to establish haploid induction systems in a variety of crops. Despite these advancements, the applicability of this technique to *B. juncea* remains uncharted. This research aims to fill the existing knowledge gap by exploring the potential of *DMP* knockout in *B. juncea*, with the goal of developing an effective haploid induction system.

In establishing haploid induction lines in *B. juncea* via *dmp*-based methods, we identified *BjuDMP1-4*. These *BjuDMPs* show high conservation in domain sequences and contain the DUF679 domain with four transmembrane regions, similar to *AtDMP8/9* [5]. Phylogenetic analysis reveals that *BjuDMP1-4* are all classified into a subclade with *AtDMP9* (Fig. 1A). *BjuDMP1* and *BjuDMP4* originate from the A subgenome. We found that they cluster with two *BnaDMPs* from *B. napus* on the A subgenome in the same branch, showing homology, which indicates that they have a common ancestor in evolution and may have similar functions. Moreover, qRT-PCR analysis indicates that the expression levels of *BjuDMP1* and *BjuDMP3* are significantly higher in the early stages of flower bud development compared to later stages. In contrast, *BjuDMP2* exhibits extremely low expression throughout the entire developmental process (Fig. 1B). Additionally, we utilized CRISPR-Cas9 technology for precise gene knockout, designed guide RNAs (gRNAs) to target the highly conserved region of the DUF679 domain within the *BjuDMPs* (Fig. 1C and D). This approach was selected based on the high homologous sequence found in this domain, which increases the likelihood of successful gene editing. The inclusion of *eGFP* serves two purposes: it aids in monitoring the results of gene editing through the detection of GFP fluorescence, and it enables the subsequent screening for haploid cells.

**Figure 1:**
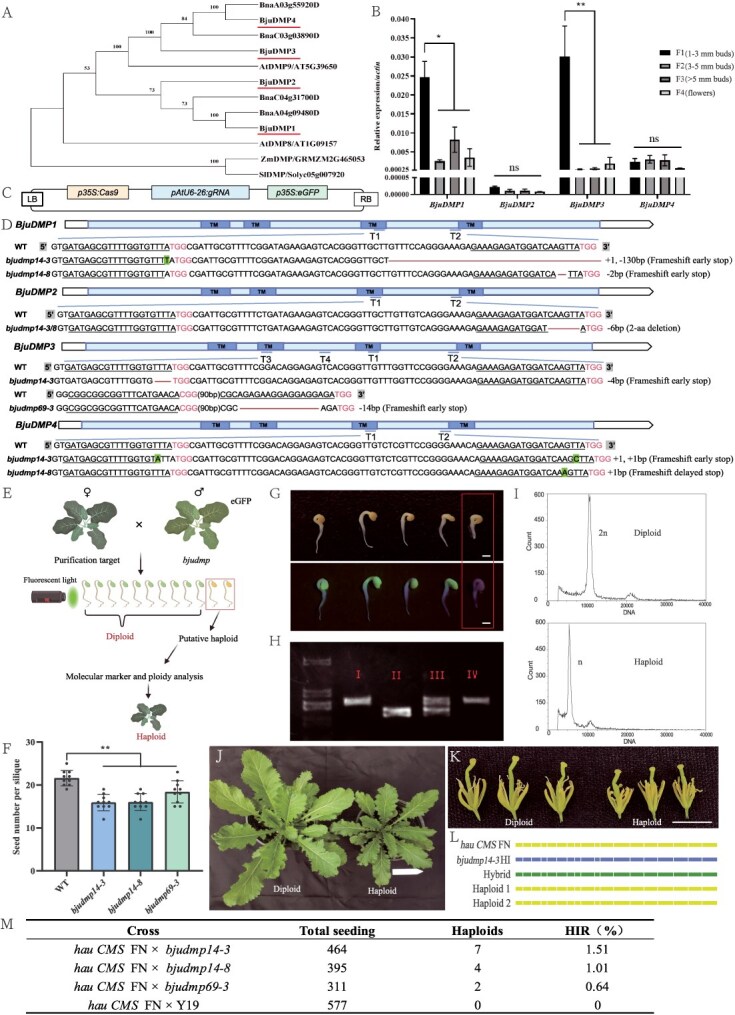
The *bjudmp* mutants trigger maternal haploid induction. **(A)** The *DMP* phylogenetic relationship tree in *Brassica juncea* (*Bju*), *Brassica napus* (*Bna*), *Arabidopsis* (*At*), *Solanum lycopersicum* (*Sl*) *and Zea mays* (*Zm*). **(B)** qRT-PCR detection of *BjuDMPs* expression across flower bud development stages. **(C)** The CRISPR-Cas9 vector with gRNAs targeting four *BjuDMPs*, and the *p35S:eGFP* selection cassettes. **(D)** A schematic diagram of the wild-type (WT) *BjuDMP* and its corresponding mutant alleles. White and light blue represent the non-coding and coding regions, respectively. The dark blue blocks correspond to the predicted transmembrane domains (TM). The blue underline indicates the regions targeted by gRNAs (T1-T4). The schematic overview is complemented by the depiction of mutant alleles, and the gRNA target sequences are underlined. **(E)** The process of inducing haploidy in mutants and the subsequent screening and identification. **(F)** The seed number per silique of selfed WT and mutants was measured (*n* = 10). **(G)** Seeds germinated under white light and fluorescent conditions. Hybrid individuals possess green fluorescence due to the paternal genotype; seeds devoid of green fluorescence within the framed area are preliminarily selected as putative haploids. **(H)** Molecular marker identification of plants lacking fluorescence. I: maternal parent, II: paternal parent, III: hybrid, IV: putative haploid. **(I)** Flow cytometric validation of ploidy in putative haploid and known diploid. **(J-K)** Comparative analysis of plants, leaves, and floral organs between haploid and diploid. Scale bars: 12 cm (J) and 1 cm (K). **(L)** Genetic background of the *bjudmp-based* HI, *hau CMS* FN, hybrid and haploid revealed by DNA markers on 18 chromosomes. **(M)** Haploid induction rate (HIR) of mutants in the progeny resulting from cross-pollination. Y19 as a control, *hau CMS* FN as the maternal line

Further to our gene editing endeavors, we identified three mutants: the quadruple-mutant line *bjudmp14-3*, the triple-mutant *bjudmp14-8* and the single-mutant line *bjudmp69-3* (Fig. 1D). Subsequent to self-pollination and segregation, we successfully obtained homozygous mutants. Frameshift mutations caused by base insertions or deletions were identified in *BjuDMP1*/*3*/*4*, and a *6-bp* deletion leading to a 2-amino acid deletion was identified in *BjuDMP2*. These mutants were subsequently utilized for further studies (Fig. 1E). A reduction in seed yield was observed in the seeds produced by self-pollination of the mutants (Fig. 1F).

Utilizing the mutant lines depicted in Fig. 1D as pollen donors for crosses with a cytoplasmic male sterility *hau CMS* FN line, which eliminates the influence of self-pollen on the induction outcomes, we screened for haploids among the harvested seeds following the strategy outlined in Fig. 1E. Haploid plants were selected and confirmed through a three-step process: first, by screening seedling hypocotyls for green fluorescence to exclude heterozygous materials exhibiting paternal fluorescence (Fig. 1G); second, by using molecular markers to eliminate heterozygous materials with double bands containing paternal genotype bands (Fig. 1H); and finally, we confirmed the haploid status of the plants by flow cytometry (Fig. 1I). Comparative analysis of *hau CMS* FN plants revealed that haploid plants, leaves, and floral organs were significantly smaller than their diploid counterparts (Fig. 1J and K). We developed 18 molecular markers, one for each chromosome, which revealed indel polymorphisms between the offspring and the maternal parent (Fig. 1L). To determine the haploid induction rate (HIR), we crossed mutants with test materials and found that HIR ranged from 0.64% to 1.51% (Fig. 1M). Among them, the quadruple mutant line *bjudmp14-3* exhibited a higher HIR, while the single mutant line *bjudmp69-3* showed a relatively lower induction rate but still possessed induction function. Given that *BjuDMP1* and *BjuDMP3* are highly expressed during early flower bud development, we hypothesize that these genes may have exhibited functional redundancy. This redundancy suggests that even when one of the genes is knocked out in certain mutant lines, the other gene may partially compensate for its function. This compensation could account for the observed differences in haploid induction rates among the various mutant lines.

In conclusion, our study successfully demonstrated the use of *BjuDMPs* to induce haploids in *B. juncea*, a major horticultural crop. This is the first application of *DMP* knockout to produce maternal haploids in *B. juncea*, simplifying the breeding process and accelerating the development of homozygous lines while reducing the labor costs. Our success suggests that similar strategies could be applied to other crops to improve breeding efficiency. These findings enrich the knowledge base on haploid induction and may inspire further research in crop improvement.

## Data Availability

The amino acid sequence of the *DMPs* presented here can be obtained from the link https://kdocs.cn/l/cpGGcwjHItGV. The additional data related to this paper may be requested from the authors.
